# Changes in smoker characteristics in England between 2008 and 2017

**DOI:** 10.1111/add.14882

**Published:** 2020-01-08

**Authors:** Claire Garnett, Ildiko Tombor, Emma Beard, Sarah E. Jackson, Robert West, Jamie Brown

**Affiliations:** ^1^ Department of Behavioural Science and Health University College London London UK

**Keywords:** Cigarette dependence, cigarette smoking, e‐cigarettes, population characteristics, smokers, smoking cessation

## Abstract

**Aims:**

At a time of declining smoking prevalence in England, it is useful to document any changes in the characteristics of smokers. This has implications for targeting tobacco control policies and interventions. This study compared the characteristics of smokers from 2008 to 2017 to assess changes in smoking and quitting patterns and socio‐demographic profile.

**Design and setting:**

Analysis of annual trends in results from repeated cross‐sectional surveys of representative samples of the population in England from 2008 to 2017.

**Participants:**

The study included 208 813 adults aged 16+.

**Measurements:**

Information was gathered on age, sex, social grade and region, cigarette consumption, cigarette dependence as measured by time to first cigarette of the day, daily smoking, smoking roll‐your‐own cigarettes, attempts to cut down, use of an e‐cigarette or nicotine replacement therapy, attempts to cut down or quit, use of support in quit attempts and whether the quit attempt was abrupt.

**Findings:**

During the period, mean daily cigarette consumption [B = –0.30, 95% confidence interval (CI) = –0.33 to −0.27] and the time to first cigarette score decreased (B = –0.03, 95% CI = –0.03 to −0.02). The proportion of smokers attempting to cut down or quit decreased (odds ratio (OR) range = 0.96–0.97, 95% CI range = 0.95–0.97). Use of behavioural support [odds ratio (OR) = 0.89, 95% CI = 0.86–0.92] or no support decreased (OR = 0.98, 95% CI = 0.96–0.99), while use of pharmacological support, including e‐cigarettes, increased (OR = 1.04, 95% CI = 1.02–1.05). There was no significant change in the difference in social grade between smokers and non‐smokers comparing 2008 with 2017. Changes in smoking and quitting behaviour were independent of changes in socio‐demographic characteristics.

**Conclusions:**

Between 2008 and 2017 in England, smokers appear to have become less dependent on cigarettes but less likely to try to quit or cut down. Of those who tried to quit, fewer used behavioural support and more used pharmacological support. The proportion from more disadvantaged backgrounds did not change significantly.

## Introduction

During the last 10 years, the prevalence of cigarette smoking in England has decreased from 21.1% in 2008 to 14.9% in 2017 (as reported by the Office for National Statistics 1). There is little analysis of representative data to describe whether important characteristics of smoking and quitting behaviour have changed over this time. This study compared nationally representative annual estimates from 2008 to 2017 of detailed smoker characteristics to provide a fuller account of how the smoking and quitting behaviour of the remaining smoking population has changed during the last 10 years. This can be used to produce appropriately targeted policies and interventions to reduce smoking prevalence further.

There have been a number of considerable changes in tobacco control policy in England since 2007, meaning that smokers' characteristics are likely to have changed in response. Smoke‐free legislation was introduced in July 2007 and was a landmark in tobacco control, making it illegal to smoke in all enclosed or substantially enclosed public areas and work‐places. Numerous other changes in tobacco control policy have occurred, including: a change in the minimum age of sale of cigarettes (October 2007) 2; licensing of nicotine replacement therapy (NRT) for harm reduction (December 2009) 3; point‐of‐sale ban (April 2012) 4; and introduction of plain packaging (May 2017) 5. Understanding the socio‐demographic profile of smokers, and how this may have changed over the last 10 years, can provide important insights to inform targeted policies and interventions. Socio‐demographic characteristics such as age, sex, socio‐economic status (SES) and region of England are associated with smoking status 6, 7, 8, 9, 10, 11, 12 and are important to consider, as socio‐economic inequalities appear to have strongly increased since during the 2000s 13. Smoking prevalence among different socio‐demographic groups has been widely reported 8, 9 although, to our knowledge, changes in the socio‐demographic characteristics of smokers have not. In addition, no direct comparison has been made between changes in smokers and non‐smokers, so it is unknown whether changes in smokers' socio‐demographic characteristics reflect those in non‐smokers in England.

The decrease in smoking prevalence and changes in tobacco control policy in England during the last 10 years are likely to have resulted in a change in the smoking and quitting behaviour of the population of smokers. There is a widely held view that as smoking prevalence decreases, progress in tobacco control becomes increasingly difficult 14, reaching a limit in terms of the minimum achievable smoking prevalence 15. This ‘hardening' hypothesis suggests that the remaining smokers are unable (high cigarette dependence) or unwilling (low motivation) to quit smoking 16. The evidence is unclear on whether the levels of cigarette dependence have changed over the last decade, and whether hardening among the population of smokers has occurred. One study found that the proportion of smokers in England between 2000 and 2010 with high cigarette dependence (indicated by time to first cigarette) increased, suggesting that ‘hardening' may have occurred 17. However, another found that the mean number of cigarettes smoked per day (another indicator of cigarette dependence) has declined as smoking prevalence falls 11, 12. There is also a limited analysis of other important smoking and quitting behaviours to provide insight into how, if at all, smokers' profiles have changed. It may also be the case that changes in smokers' smoking and quitting behaviour are driven by changes in socio‐demographic characteristics. There is currently an incomplete picture of how smokers' characteristics have changed over the last decade in England.

This study used a large, nationally representative, cross‐sectional population survey in England to compare the smoking and quitting behaviour of smokers over 10 years from 2008 to 2017.

### Research questions

1. Has the smoking and quitting behaviour of adult smokers changed from 2008 to 2017?
2. Are changes in socio‐demographic characteristics of adult smokers (comparing 2017 with 2008) different from changes in the non‐smoker population?
3. Are changes in smoking and quitting behaviour from 2008 to 2017 independent of changes in socio‐demographic characteristics among smokers?

## Methods

### Study design

Data were collected as part of the Smoking Toolkit Study (STS), an ongoing, monthly population survey in England. The STS consists of cross‐sectional monthly household surveys of representative samples of approximately 1800 adults (aged 16+) in England 18. The sampling is a hybrid of random probability and simple quota—England is split into 171 356 areas (consisting of approximately 300 households each) stratified according to a geo‐demographic analysis of the population and then areas are randomly allocated to interviewers who conduct interviews within that area until the quota is fulfilled. Response rates cannot be calculated because there is no definite gross sample, with units fulfilling the criteria of the quota being interchangeable.

### Study population

For this study, data were used from respondents to the survey between January 2008 and December 2017 (inclusive).

### Measures

All respondents were asked questions assessing their socio‐demographic characteristics: age (in banded years), sex (female/male), social grade as a measure of SES [dichotomized into high (ABC1: managerial, professional and intermediate occupations)/low (C2DE: skilled, semi‐skilled, unskilled manual and lowest‐grade worked or unemployed)], region in England (North/Central/South) and smoking status (current smoker/non‐smoker: including recent ex‐smoker, long‐term ex‐smoker or never smoker).

Respondents who were current smokers were also asked questions assessing their smoking and quitting behaviour: number of cigarettes smoked per day (indicator of cigarette dependence); time to first cigarette (indicator of cigarette dependence: more than 60 minutes/30–60 minutes/6–30 minutes/within 5 minutes 19); daily versus non‐daily cigarette smoking; smoking any roll‐your‐own cigarettes versus none; whether or not the respondent was currently cutting down (‘Are you currently trying to cut down on how much you smoke but not currently trying to stop?'), using an e‐cigarette or NRT (for any purpose) or had tried to quit in the past year (‘How many serious attempts to stop smoking have you made in the last 12 months?', with responses dichotomized into yes/no).

Current smokers who had tried to quit in the past year were asked whether their most recent quit attempt was abrupt (‘Did you cut down the amount you smoked before trying to stop completely at your most recent serious quit attempt?': cut down first/stopped without cutting down). Current smokers were asked to select from a list of options what they had used to help them stop smoking during their most recent serious quit attempt, which we categorized into pharmacological (i.e. NRT, varenicline, bupropion or e‐cigarettes), face‐to‐face behavioural support and nothing).

### Analyses

All analyses were conducted in R studio (version 1.0.153). Data were weighted using the rim (marginal) weighting technique to match an English population profile on the dimensions of age, social grade, region, tenure, ethnicity and working status within sex.

The protocol and analysis plan were pre‐registered on the Open Science Framework (https://osf.io/skphm). An amendment was made to the protocol with data being used from 2008 to 2017 due to substantial missing data for an important outcome variable (proportion of roll‐your‐own smokers) in 2007. Three outcome variables were not collected for every wave during 2012 and 2013 (use of e‐cigarettes: 5.5% missing data; use of NRT: 5.5% and cutting down: 9.9%). Multiple imputation was used to impute the missing values for the three outcome variables (use of e‐cigarettes, use of NRT and cutting down) using all other variables as predictors (complete cases only). Five imputed data sets were created 20, analysed individually and then the results combined using Rubin's rules 21, to produce the reported pooled estimates. Complete case analysis was then conducted for all other variables of interest (1.2% of respondents had missing data on a variable of interest and were removed from the analytical sample).

Descriptive statistics [means ± standard deviations (SDs) or % (*n*), as appropriate] were used to report the variables included in the analyses (socio‐demographic characteristics and smoking and quitting behaviour). Generalized linear modelling (linear or logistic, as appropriate) was used to examine whether smoking and quitting behaviour among current smokers (outcome variable) had changed over time (predictor variable). These models were adjusted for socio‐demographic characteristics to assess whether any changes in smoking and quitting behaviour among current smokers over time were independent of changes in socio‐demographic characteristics. Generalized linear modelling (linear, logistic or multinomial logistic, as appropriate) was used to examine the associations between socio‐demographic characteristics (outcome variable) and year (2008 versus 2017), smoking status (current smoker versus non‐smoker) and their interaction (predictor variables, entered into the model separately).

## Results

An unweighted total of 208 813 adults aged 16+ responded to the survey between January 2008 and December 2017. Of these, 19.9% were current smokers [95% confidence interval (CI) = 19.8–20.1%], and an unweighted total of 41 610 respondents were included in the analyses assessing whether smoking and quitting behaviour of adult smokers has changed from 2008 to 2017 (inclusive). Supporting information, Fig. S1 shows the change in smoking prevalence from 2008 to 2017.

An unweighted total of 38 868 adults aged 16+ were included in the analysis assessing whether changes in socio‐demographic characteristics of adult smokers (comparing 2008 with 2017) were different from changes in the non‐smoker population; of these, 7348 were current smokers and 31 520 were non‐smokers. Table 1 reports weighted descriptive statistics on the smoking and quitting behaviour of adult smokers in England for 2008 and 2017.

**Table 1:** Smoking and quitting behaviour of current smokers in England for 2008 and 2017.

Smoking and quitting behaviour	Year	
	2008	2017
Cigarettes per day, mean (SD)	13.6 (8.69)	10.9 (8.13)
Time to first cigarette score, mean (SD)^a^	1.5 (1.08)	1.2 (1.08)
Non‐daily smoker, % (*n*)	9.1 (369)	13.4 (437)
Smoking roll‐your‐own, % (*n*)	35.3 (1428)	50.7 (1661)
Currently cutting down, % (*n*)	56.1 (2268)	47.9 (1569)
Current use of e‐cigarette, % (*n*)	0 (0)	18.7 (611)
Current use of NRT, % (*n*)	17.3 (700)	9.8 (320)
Tried to quit in past year, % (*n*)	37.0 (1497)	29.9 (978)
Most recent quit attempt was abrupt, % (*n*)^b^	56.8 (850)	48.5 (474)
*Use of support during the most recent quit attempt, %* (*n*)^b^		
Pharmacological^c^	50.8 (760)	54.0 (527)
Behavioural^d^	5.8 (87)	3.3 (32)
None	46.9 (702)	45.3 (442)

a Excluding data from the first three waves of 2008 (waves 16–18, inclusive) as the lowest two scoring responses for this question were not distinguished. Time to first cigarette scoring (0: > 60 minutes; 1: 30–60 minutes; 2: 6–30 minutes; 3: < 5 minutes).

b Question only asked of those who tried to quit in past year.

c Pharmacological: use of any prescription medication, alternative nicotine product or e‐cigarette.

d Behavioural: use of any face‐to‐face behavioural support [National Health Service (NHS)/non‐NHS stop smoking group/one‐to‐one]. NRT = nicotine replacement therapy; SD = standard deviation.

### Has the smoking and quitting behaviour of adult smokers changed from 2008 to 2017?

Table 2 reports the associations between year (continuous from 2008 to 2017) and smoking and quitting behaviour of adult smokers in England. Supporting information, Table S1 reports descriptive statistics on the smoking and quitting behaviour for each year from 2008 to 2017. The mean number of cigarettes smoked per day and the time to the first cigarette score (higher scores represent a shorter time) both decreased, indicating a decrease in cigarette dependence. The proportion who were non‐daily smokers, smoking roll‐your‐own cigarettes and currently using e‐cigarettes increased. The proportion of smokers currently cutting down, currently using NRT and who tried to quit in the past year decreased. Of smokers who tried to quit in the past year, the proportion whose most recent quit attempt was abrupt decreased, as did the use of behavioural support and no support during their most recent quit attempt. The proportion of smokers using pharmacological support during their most recent quit attempt increased.

**Table 2:** Association between year (continuous from 2008 to 2017) and smoking and quitting behavior.

Smoking and quitting behaviour	Unadjusted		Adjusted for socio‐demographic characteristics	
	B (95% CI)	P	B (95% CI)	P
Cigarettes per day	−0.29 (−0.32, −0.26)	< 0.001	−0.30 (−0.33, −0.27)	< 0.001
Time to first cigarette score^a^	−0.03 (−0.03, −0.02)	< 0.001	−0.03 (−0.03, −0.02)	< 0.001
	*OR (95% CI)*	*P*	OR (95% CI)	*P*
Non‐daily smoker	1.03 (1.01, 1.04)	< 0.001	1.03 (1.02, 1.04)	< 0.001
Smoking roll‐your‐own	1.07 (1.07, 1.08)	< 0.001	1.08 (1.07, 1.09)	< 0.001
Currently cutting down	0.96 (0.95, 0.97)	< 0.001	0.96 (0.95, 0.97)	< 0.001
Current use of e‐cigarette	1.45 (1.43, 1.46)	< 0.001	1.45 (1.43, 1.47)	< 0.001
Current use of NRT	0.90 (0.89, 0.91)	< 0.001	0.90 (0.89, 0.91)	< 0.001
Tried to quit in past year	0.96 (0.96, 0.97)	< 0.001	0.97 (0.96, 0.97)	< 0.001
Most recent quit attempt was abrupt^b^	0.96 (0.94, 0.97)	< 0.001	0.96 (0.95, 0.97)	< 0.001
Use of support during the most recent quit attempt^b^				
Pharmacological^c^	1.04 (1.02, 1.05)	< 0.001	1.04 (1.02, 1.05)	< 0.001
Behavioural^d^	0.89 (0.86, 0.92)	< 0.001	0.89 (0.86, 0.92)	< 0.001
None	0.97 (0.96, 0.99)	< 0.001	0.98 (0.96, 0.99)	< 0.001

a Excluding data from the first three waves of 2008 (waves 16–18, inclusive) as the lowest two scoring responses for this question were not distinguished. Time to first cigarette scoring (0: > 60 minutes; 1: 30–60 minutes; 2: 6–30 minutes; 3: < 5 minutes).

b Question only asked in those who tried to quit in past year.

c Pharmacological: use of any prescription medication, alternative nicotine product or e‐cigarette

d Behavioural: use of any face‐to‐face behavioural support [National Health Service (NHS)/non‐NHS stop smoking group/one‐to‐one]. OR = odds ratio; CI = confidence interval; NRT = nicotine replacement therapy.

### Were the changes in socio‐demographic characteristics of adult smokers (comparing 2008 with 2017) different from changes in the non‐smoker population?

Table 3 reports descriptive statistics for the socio‐demographic characteristics of current smokers and non‐smokers for 2008 and 2017. Supporting information, Table S2 reports smoking prevalence in 2008 and 2017 by socio‐demographic characteristics, for ease of comparing these data with other national surveys (e.g. Health Survey for England 9 and Office for National Statistics 8).

**Table 3:** Socio‐demographic characteristics by smoking status and year

	Current smoker		Non‐smoker	
	2008	2017	2008	2017
Age, mean (SD)	40.0 (15.85)	41.5 (16.33)	47.6 (19.08)	48.2 (19.25)
Sex, % female (*n*)	49.6 (2005)	47.9 (1568)	52.0 (7700)	51.6 (8640)
Social grade, % ABC1 (*n*)	38.3 (1549)	38.4 (1257)	60.2 (8921)	58.4 (9783)
Region, % (*n*)				
North	35.4 (1431)	32.0 (1047)	26.8 (3966)	27.5 (4613)
Central	31.5 (1275)	29.4 (962)	30.9 (4582)	30.4 (5088)
South	33.1 (1340)	38.6 (1264)	42.3 (6259)	42.1 (7049)

SD = standard deviation.

Table 4 reports the association of socio‐demographic characteristics with year (2008 versus 2017) and smoking status (current smoker versus non‐smoker). Age was associated with year and smoking status. There was also a two‐way interaction, which indicated that changes in age between 2008 and 2017 were different between smokers and non‐smokers. When stratified by smoking status, there was a significant positive association between age and year among both smokers and non‐smokers. Smokers were, on average, 1.53 years older in 2017 (95% CI = 0.75–2.30, *P* < 0.001) compared with 2008, while non‐smokers were 0.56 years older (95% CI = 0.12–1.01, *P* = 0.013). There was a higher proportion of females and social grade ABC1 among non‐smokers. There was no two‐way interaction between year and smoking status for either variable, indicating that changes between 2008 and 2017 in the proportion of females and in social grade were similar between smokers and non‐smokers. Smoking status was associated with both Central and South (versus North) regions. There was a significant two‐way interaction between year and smoking status on South region, indicating that the magnitude of association over time between South versus North was dependent on smoking status. Stratified by smoking status, there was a significant increase in the proportion of smokers from the South (versus North) between 2008 and 2017 [odds ratio (OR) = 1.29, 95% CI = 1.15–1.44, *P* < 0.001], but no significant change in the proportion of non‐smokers from the South versus North over time (OR = 0.97, 95% CI = 0.92–1.02, *P* = 0.245). There was a similar pattern of results when conducting *post‐hoc* sensitivity analyses comparing 2008 with 2016 and 2009 with 2017.

**Table 4:** Association between year (2008 versus 2017) and smoking status (current versus non‐ smoker) with socio‐demographic characteristics.

	Year (2008^a^ versus 2017)		Smoking status (current smoker^a^ versus non‐smoker)		Interaction (year × smoking status)	
	B (95% CI)	P	B (95% CI)	P	B (95% CI)	P
Age, mean (SD)	1.11 (0.72, 1.50)	< 0.001	7.26 (6.82, 7.70)	< 0.001	−0.96 (−1.86, −0.07)	0.034
	*OR (95% CI)*	*P*	OR (95% CI)	*P*	OR (95% CI)	*P*
Sex (female^a^ versus male)	1.02 (0.98, 1.06)	0.355	0.89 (0.84, 0.94)	< 0.001	0.95 (0.85, 1.06)	0.375
Social grade (ABC1^a^ versus C2DE)	1.02 (0.97, 1.06)	0.463	0.43 (0.40, 0.45)	< 0.001	1.08 (0.97, 1.21)	0.157
Region, % (*n*)						
North^a^						
Central	0.98 (0.94, 1.04)	0.566	1.25 (1.17, 1.33)	< 0.001	0.93 (0.81, 1.05)	0.245
South	1.04 (0.99, 1.09)	0.089	1.48 (1.39, 1.57)	< 0.001	0.75 (0.66, 0.85)	< 0.001

a Reference group. SD = standard deviation; OR= odds ratio; CI = confidence interval.

### Were changes in smoking and quitting behaviour from 2008 to 2017 independent of changes in socio‐demographic characteristics among smokers?

Table 2 reports the associations between year and smoking and quitting behaviour, adjusted for socio‐demographic characteristics. All the changes in smoking and quitting behaviour among smokers in England from 2008 to 2017 were independent of changes in their socio‐demographic characteristics.

## Discussion

### Summary of findings

Smoking and quitting behaviour among smokers in England has changed between 2008 and 2017, independent of any changes in their socio‐demographic characteristics. Cigarette dependence appears to have decreased, as indicated by a reduction in the average number of cigarettes smoked daily and longer time to the first cigarette. There were increases in the proportion of non‐daily smokers and the proportion smoking roll‐your‐own cigarettes. Current use of NRT decreased, while current use of e‐cigarettes increased. The proportion of smokers cutting down or making a quit attempt in the past year decreased. Of those making a quit attempt, the proportion making an abrupt quit attempt decreased. Use of no support and use of behavioural support decreased during the most recent quit attempt, while use of pharmacological support increased.

The reduction in the average number of cigarettes smoked daily and longer time to the first cigarette among current smokers indicate a decrease in cigarette dependence among smokers in England from 2008 to 2017. This contradicts the ‘hardening' hypothesis, which states that as smoking prevalence falls, levels of cigarette dependence would increase 16. Lower levels of cigarette dependence consistently predict greater quit success 22, suggesting that there is the potential to further reduce smoking prevalence in the English population. Other measures of dependence (e.g. strength of urges to smoke) may have shown a different pattern of results. The increase in the proportion of non‐daily smokers may relate to this finding that cigarette dependence appears to have decreased in England during the last 10 years, as non‐daily smokers regularly engage in voluntary abstinence. However, caution should be taken, as it was found that non‐daily smokers in the United States experienced difficulty in quitting 23, despite not showing other signs of dependence 24.

The proportion smoking roll‐your‐own cigarettes increased from 2008 to 2017. This is probably related to progressive tax increases on tobacco in the United Kingdom that have resulted in roll‐your‐own cigarettes often being substantially less expensive than manufactured cigarettes 25. Smokers' current use of NRT decreased, while current use of e‐cigarettes increased drastically. There is evidence that NRT can help smokers who make a quit attempt to increase their chances of successful cessation 26, 27, and evidence from randomized controlled trials suggests that the use of e‐cigarettes increases smoking cessation 28, 29, 30. E‐cigarette users in England also report reasons such as to avoid cigarettes, enjoyment, to relieve stress or anxiety and to improve health 31. The decline in use of NRT does not appear to be a consequence of the increase in e‐cigarette use 32. The proportion of smokers currently cutting down or attempting to quit decreased. Access to stop smoking services is an important factor that determines whether smokers attempt to cut down or quit smoking. Access to services has decreased due to recent budget cuts in approximately half of local authorities 33, 34, 35. This is particularly concerning, as these services successfully reach disadvantaged smokers 36 and the percentage of current smokers of low social grade has remained disproportionally high (61.7% in 2008 and 61.6% in 2017) even as smoking prevalence has fallen. This indicates a lack of progress by existing interventions and policies in tackling these disparities in smoking prevalence and highlights the need to reinstate access to effective services that can successfully reach disadvantaged smokers. Targeted interventions and policies are needed for disadvantaged smokers, who comprise the majority of the smoker population, to progress both in further reducing overall smoking prevalence and in reducing disparities. The 2017 ‘Tobacco Control Plan' for England aims to reduce the inequality gap in smoking prevalence and details specific actions to take, such as supporting local councils with high smoking rates in their evidence‐based local tobacco control plans 37.

Of those smokers making a quit attempt in the past year, the proportion making an abrupt quit attempt and using behavioural support decreased from 2008 to 2017. This is concerning, as increased quit success is associated with both abrupt quit attempts 38, 39 and use of behavioural support 26, 38. The use of pharmacological support, which is known to improve the chances of a successful quit attempt 38, increased; this is likely to be due, in part, to the rapid rise in prevalence of e‐cigarette use. However, more encouragingly, the proportion of smokers using no aids during their most recent quit attempt decreased. It is important for future research to understand what the implications of these changes are for rates of quit success.

Changes in smokers' sex and social grade between 2008 and 2017 were broadly representative of changes in the non‐smoker population. Smokers and non‐smokers were both significantly older in 2017 compared with 2008, although smokers had a significantly larger increase in age (on average, 1.53 years) compared with non‐smokers (0.56 years). This may be due, in part, to the policy change in October 2007 which increased the legal age of sale to 18 years and, in turn, resulted in a greater fall in smoking prevalence of 16–17‐year‐olds 2. There was an increase in the proportion of smokers from the South compared with the North of England (5.5 percentage points) between 2008 and 2017, while the proportion of non‐smokers in the South has remained fairly stable. This change may be driven by other changes in socio‐demographic profiles over time (e.g. with immigration, differential investment), suggesting that by addressing the most at‐risk smokers across the country and allocating resources to deprived areas, the variation in smoking rates and health inequalities can be reduced 40, 41.

### Strengths and limitations

This is the first large population study, to our knowledge, to evaluate changes in smoking and quitting behaviour over a 10‐year period in England. There are existing studies evaluating the changes in constructs of the hardening hypothesis (i.e. nicotine dependence, motivation to quit, quit rates) in New Zealand 42 and the United States 43, which found no evidence of hardening at the population level over time. We are not aware of any studies in other countries assessing the equivalent changes in smoking and quitting patterns and socio‐demographic profile over a 10‐year period. The findings may not generalize to other counties, as England could be considered unique in its extensive tobacco control policies and relatively liberal attitude towards tobacco control and harm reduction with the legalization of e‐cigarettes. It provides a detailed analysis of how smoking and quitting behaviours have changed as the prevalence of smoking has decreased, and describes how smokers' socio‐demographic characteristics have changed since 2007. The current study focuses on the socio‐demographic characteristics of smokers, rather than smoking prevalence for different socio‐demographic characteristics, which is how data tend to be reported. This allowed a direct comparison between the change over time in smokers and non‐smokers' socio‐demographic characteristics.

Although this paper had several advantages, there were also a number of limitations. First, this paper only assessed linear associations between time (in years) and smoking characteristics. It would be of interest to conduct a trend analysis in order to assess whether non‐linear, perhaps quadratic or cubic, trends provide a better fit. However, this would require a longer series and was beyond the scope of the current study. Another limitation is that there was no measure for motivation to quit—a separate construct in the ‘hardening' hypothesis—included in the STS from the start of the current study period. This meant that no analysis could be conducted of how motivation to quit changed, if at all, over time that would inform the conclusions around the ‘hardening' hypothesis. There was a reliance on recall data for variables relating to quitting behaviour, which involved recall of the past year, introducing scope for bias. This study underestimates overall smoking prevalence in England 44, as a greater proportion of smokers were excluded for having missing data compared with non‐smokers (smokers were required to have complete cases for more variables). However, smoking prevalence was not an outcome of this study, and its underestimation did not affect the analysis of change in smoking and quitting behaviour over time.

## Conclusions

Smokers' smoking and quitting behaviour have changed substantially in England between 2008 and 2017 as smoking prevalence has decreased. These changes were not driven by any changes in smokers' socio‐demographic characteristics. The proportion of smokers of low social grade remains high but unchanged, indicating a lack of progress in reducing the social gradient in smoking prevalence and highlighting the need for targeted interventions and policies. Smokers smoke fewer cigarettes per day and the time to their first cigarette has increased, indicating that cigarette dependence has decreased, in contrast with concerns that falls in smoking prevalence leave behind a more dependent, ‘hardened' smoking population. More smokers are non‐daily smokers and smoke roll‐your‐own cigarettes. Attempts to quit and cut down have decreased, as has use of behavioural support such as National Health Service (NHS) stop‐smoking services, highlighting the need to reinstate and improve easy to access effective services. Of those smokers making quit attempts, fewer use no support while more use pharmacological support, due probably to the rapid rise in the prevalence of e‐cigarette use.

## Declaration of interest

C.G., I.T. and S.J. have no competing interests. J.B. and E.B. have received unrestricted research grants from Pfizer related to smoking cessation. R.W. has received research funding and undertaken consultancy for companies that manufacture smoking cessation medications.

## Supporting information


**Figure S1** Graph tox` show change in smoking prevalence from 2008 to 2017.

**Table S1** Smoking and quitting behaviour of current smokers in England for 2008 and 2017
Supplementary Table 2: Smoking prevalence by socio‐demographic characteristics and year.
