# Supplementary figures and images for: Changes in smoker characteristics in England between 2008 and 2017
Source: Addiction. 2020 Jan 8;115(4):748–56. doi: 10.1111/add.14882 (PMC7079121; doi:10.1111/add.14882)

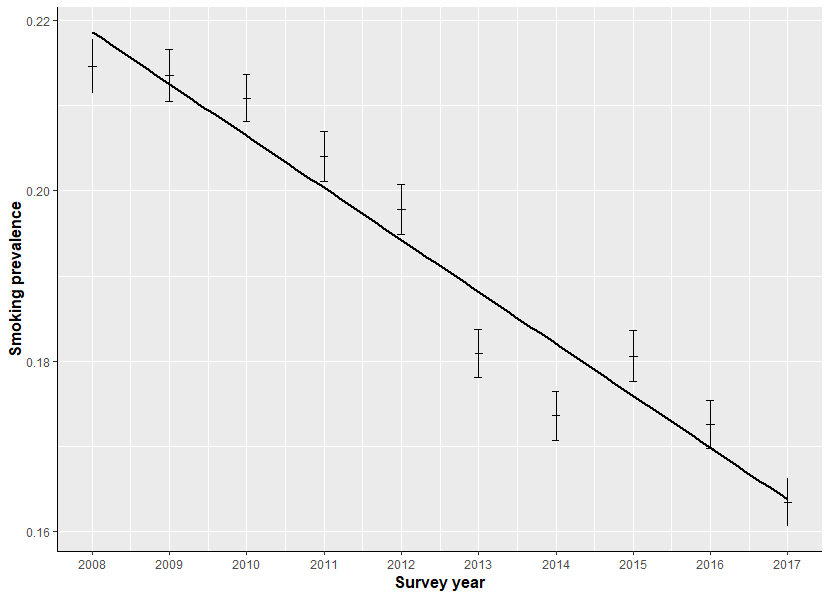


Supplementary Figure 1: Graph to show change in smoking prevalence from 2008 to 2017

Supplement: Supplementary file 1 — Figure S1 Graph tox` show change in smoking prevalence from 2008 to 2017. [file ADD-115-748-s001.docx]
