# Supplementary material for: Changes in smoker characteristics in England between 2008 and 2017
Source: Addiction. 2020 Jan 8;115(4):748–56. doi: 10.1111/add.14882 (PMC7079121; doi:10.1111/add.14882)
Supplement: Supplementary file 2 — Table S1 Smoking and quitting behaviour of current smokers in England for 2008 and 2017 Supplementary Table 2: Smoking prevalence by socio‐demographic characteristics and year. [file ADD-115-748-s002.docx]

Supplementary Table 1: Smoking and quitting behaviour of current smokers in England for 2008 and 2017

| Smoking and quitting behaviour | Year | | | | | | | | | |
| --- | --- | --- | --- | --- | --- | --- | --- | --- | --- | --- |
|  | 2008 | 2009 | 2010 | 2011 | 2012 | 2013 | 2014 | 2015 | 2016 | 2017 |
| Cigarettes per day, Mean (SD) | 13.6 (8.69) | 13.1 (8.22) | 12.8 (8.41) | 12.4 (8.07) | 12.2 (8.40) | 11.8 (8.01) | 11.6 (8.25) | 11.3 (8.13) | 11.3 (8.21) | 10.9 (8.13) |
| Time to first cigarette score, Mean (SD)^a^ | 1.5 (1.08) | 1.5 (1.08) | 1.4 (1.10) | 1.4 (1.09) | 1.4 (1.08) | 1.3 (1.08) | 1.4 (1.09) | 1.3 (1.10) | 1.3 (1.08) | 1.2 (1.08) |
| Non-daily smoker, % (n) | 9.1 (369) | 11.6 (522) | 11.3 (588) | 8.7 (389) | 9.8 (413) | 12.3 (484) | 10.6 (363) | 11.9 (422) | 11.2 (390) | 13.4 (437) |
| Smoking roll-your-own, % (n) | 35.3 (1428) | 37.8 (1699) | 39.1 (2035) | 38.7 (1720) | 41.9 (1762) | 47.4 (1860) | 45.8 (1570) | 47.9 (1700) | 48.6 (1686) | 50.7 (1661) |
| Currently cutting down, % (n) | 56.1 (2268) | 53.6 (2408) | 52.4 (2733) | 51.1 (2272) | 51.3 (2158) | 51.8 (2035) | 47.9 (1641) | 47.3 (1682) | 45.2 (1567) | 47.9 (1569) |
| Current use of e-cigarette, % (n) | 0.0 (0) | 0.2 (9) | 0.3 (14) | 2.6 (114) | 8.9 (376) | 17.5 (689) | 19.8 (679) | 21.4 (761) | 20.5 (710) | 18.7 (611) |
| Current use of NRT, % (n) | 17.3 (700) | 19.5 (878) | 17.7 (923) | 16.1 (715) | 15.1 (637) | 15.5 (610) | 11.0 (377) | 9.3 (331) | 8.5 (294) | 9.8 (320) |
| Tried to quit in past year, % (n) | 37.0 (1497) | 34.4 (1545) | 33.1 (1723) | 30.9 (1373) | 30.6 (1289) | 34.0 (1335) | 32.5 (1114) | 28.1 (999) | 26.6 (923) | 29.9 (978) |
| Most recent quit attempt was abrupt, % (n)^b^ | 56.8 (850) | 55.7 (860) | 54.1 (927) | 53.2 (721) | 50.3 (645) | 51.1 (681) | 50.7 (562) | 46.9 (467) | 47.3 (436) | 48.5 (474) |
| *Use of support during the most recent quit attempt^b^* |  |  |  |  |  |  |  |  |  |  |
| Pharmacological^c^ | 50.8 (760) | 48.5 (748) | 49.9 (856) | 52.6 (713) | 55.1 (706) | 58.1 (774) | 57.7 (640) | 56.9 (568) | 56.5 (521) | 54.0 (527) |
| Behavioural^d^ | 5.8 (87) | 6.7 (104) | 5.9 (102) | 3.7 (50) | 5.1 (66) | 3.2 (42) | 3.0 (34) | 2.7 (26) | 2.3 (21) | 3.3 (32) |
| None | 46.9 (702) | 49.1 (758) | 46.9 (803) | 45.4 (615) | 42.1 (540) | 40.6 (541) | 41.2 (457) | 42.5 (424) | 42.7 (394) | 45.3 (442) |

^a^ excluding data from the first 3 waves of 2008 (wave 16 – 18, inclusive) as the lowest two scoring responses for this question were not distinguished. Time to first cigarette scoring (0: >60 minutes; 1: 30-60 minutes; 2: 6-30 minutes; 3: <5 minutes)

^b^ question only asked in those who tried to quit in past year

^c^ pharmacological: use of any prescription medication, alternative nicotine product or e-cigarette ^d^ behavioural: use of any face-to-face behavioural support (NHS/non-NHS stop smoking group/one-to-one)

Supplementary Table 2: Smoking prevalence by sociodemographic characteristics and year

|  | Smoking prevalence, % (n) | |
| --- | --- | --- |
|  | 2008 | 2017 |
| All | 21.5 (4046) | 16.3 (3273) |
| Age |  |  |
| 16-24 | 28.8 (849) | 20.8 (595) |
| 25-34 | 27.5 (831) | 22.3 (748) |
| 35-44 | 25.3 (900) | 17.6 (572) |
| 45-54 | 22.4 (667) | 17.7 (611) |
| 55-64 | 17.0 (459) | 14.8 (412) |
| 65+ | 9.3 (339) | 7.8 (334) |
| Sex |  |  |
| Female | 20.7 (2005) | 15.4 (1568) |
| Male | 22.3 (2040) | 17.4 (1705) |
| Social grade |  |  |
| ABC1 | 14.8 (1549) | 11.4 (1258) |
| C2DE | 29.8 (2496) | 22.4 (2015) |
| Region |  |  |
| North | 26.5 (1431) | 18.5 (1047) |
| Central | 21.8 (1275) | 15.9 (962) |
| South | 17.6 (1340) | 15.2 (1264) |
